# Gender-wise comparison of oral health quality of life and its relationship with oral health parameters among elderly from Wroclaw, south-west Poland

**DOI:** 10.1371/journal.pone.0259286

**Published:** 2021-11-03

**Authors:** Katarzyna Skośkiewicz-Malinowska, Urszula Kaczmarek, Barbara Malicka

**Affiliations:** Department of Conservative Dentistry with Endodntics, Wroclaw Medical University, Wroclaw, Poland; All India Institute of Medical Sciences, INDIA

## Abstract

**Background:**

In recent years, there has been an increase of aging population with longer life expectancy in females. This study aims to compare some oral health parameters and quality of life in the elderly.

**Methods:**

The survey involved 500 urban residents (Wroclaw, Poland) aged 65 and older, of both gender. Socio-demografic data were assessed by self-reported questionnaire. Clinical examination included oral health assessment by the World Health Organization criteria with extension and oral dryness (Chalacombe scale). Quality of Life (QoL) was evaluated using Euro-Quality of Life, Oral Health Impact Profile-14 and Patient Health Questionnaire-9, which were validated for the Polish population. The relationship strength between psychometric scale scores and sociodemographic and clinical factors was determined by calculating Spearman’s linear correlation coefficient values and regression coefficient values.

**Results:**

There was no gender-wise differences in oral health parameters, except for a higher number of decayed teeth in males (DT 1.9±3.2 vs 1.2±2.4; p = 0.34). Oral dryness was diagnosed significantly more frequently in females then males (36.9% vs. 25.5%; p = 0.076). The males were significantly more likely to have high treatment needs (36.1% vs. 26.9%; p = 0.032) and they required urgent dental treatment (7.2% vs. 2.8%; p = 0.022). There were no significant differences in terms of QoL evaluated by EQ-5D, EQ-5D VAS or OHIP-14 questionnaires between males and females (0.832±0.194 vs 0.855±0.197, 67.9±10.9 vs 66.1±18.6, 7.2±12.9 vs 8.5±14.0, respectively; p > 0.05). However, females presented the higher severity of depressive symptoms measured by the PHQ-9 questionnaire (4.0±4.1 vs. 2.8±3.8; p<0.001).

**Conclusion:**

It can be concluded that the independent predictors which significantly affect the high QoL scores on the EQ-5D scale were found to be female gender, age below 75, high or middle income, independence in daily life, a low number of comorbidities, lack of oral treatment needs.

## Introduction

Quality of life is a broad, multifaceted concept that is of interest not only in the field of psychology, philosophy, and also medicine. The concept of quality of life (QoL) encompasses several important criteria that have a significant impact on the evaluation of its level, including physical, material, social, emotional well-being as well as satisfaction with one’s own productivity. A person’s QoL is also affected by their health and psychological well-being. This is because illness can affect a sense of QoL on many simultaneous levels [1–3].

In recent years, there has been an increase in the number of ageing population, especially in highly developed countries. It is conditioned by the increasing length of life as a result of civilisation progress with a simultaneous decline in births, resulting in an increase in a percentage of the population of the elderly compared to younger persons. Life expectancy is a demographic data element that is frequently used and analysed in all countries. It represents the average life expectancy at birth and it results from the overall health of a country’s population. Women have a longer life expectancy compared to men. The reason for this variation is not fully explained; biological factors have been suggested, as well as the fact that men more often undertake work of a particularly hazardous nature or pursue riskier activities [4, 5]. Currently in Poland, the average life expectancy for women is 80.2 years and for men– 71.5 years (a difference of 8.7 years) [6, 7]. The feature of demographic ageing in Poland is the feminization of old age, expressed in the predominance of elderly women. Women’s ageing is associated with more frequent living alone and with significantly lower income, it is difficult for widowed women to meet their needs independently. Hence the purposefulness of the conducted research was the need to analyze the implications of the feminization and singularization phenomena of oral health and quality of life among elderly in Poland [7].

As overall health and oral health deteriorate with age, the World Health Organization (WHO) has for many years advocated for actions on health promotion for the elderly population as a target group. Hence, active ageing is about healthy ageing, active participation in society, fulfillment in professional life and independence in daily life [8]. As regards scientific grounds for longer survival expectancy for women, the presence or absence of differences between individuals of both gender in terms of basic oral health parameters, as well as in terms of perception of QoL, overall health and emotional state, seems to be an interesting issue. Although the assessment of basic oral health parameters seems certain by routinely used clinical indicators of hard tissue diseases and periodontal diseases, the evaluation of QoL and selection of appropriate tools may be more problematic. A vast number of standardised questionnaires were developed to evaluate QoL, with particular emphasis on general health [8–10].

This study aims to compare oral health parameters and conduct a psychometric assessment using standardised inventories of QoL in a group of the Polish urban residents of both gender aged 65 and more. The null hypothesis was there was no difference beetwen males and females in the studied parameters.

## Materials and methods

### Study design

The presented observational study was carried out as part of the project "Oral Health and Quality of Life in Old Age: A Cross-Sectional Pilot Project in Germany and Poland" in cooperation between Municipal Council and the University Hospital Carl Gustav Carus in Dresden, Germany. The Polish population was examined over the period of 24 months after obtaining the consent of the Bioethics Committee of Wroclaw Medical University. The study was supported by Municipal Council resources as part of the project "Oral Health and Quality of Life of Elderly Residents of Wroclaw) (financial agreement P/ZJU/1/2015-2017).

The STROBE guidelines (Strengthening the Reporting of Observational Studies in Epidemiology were followed [11].

### Participants

**Fig 1:**
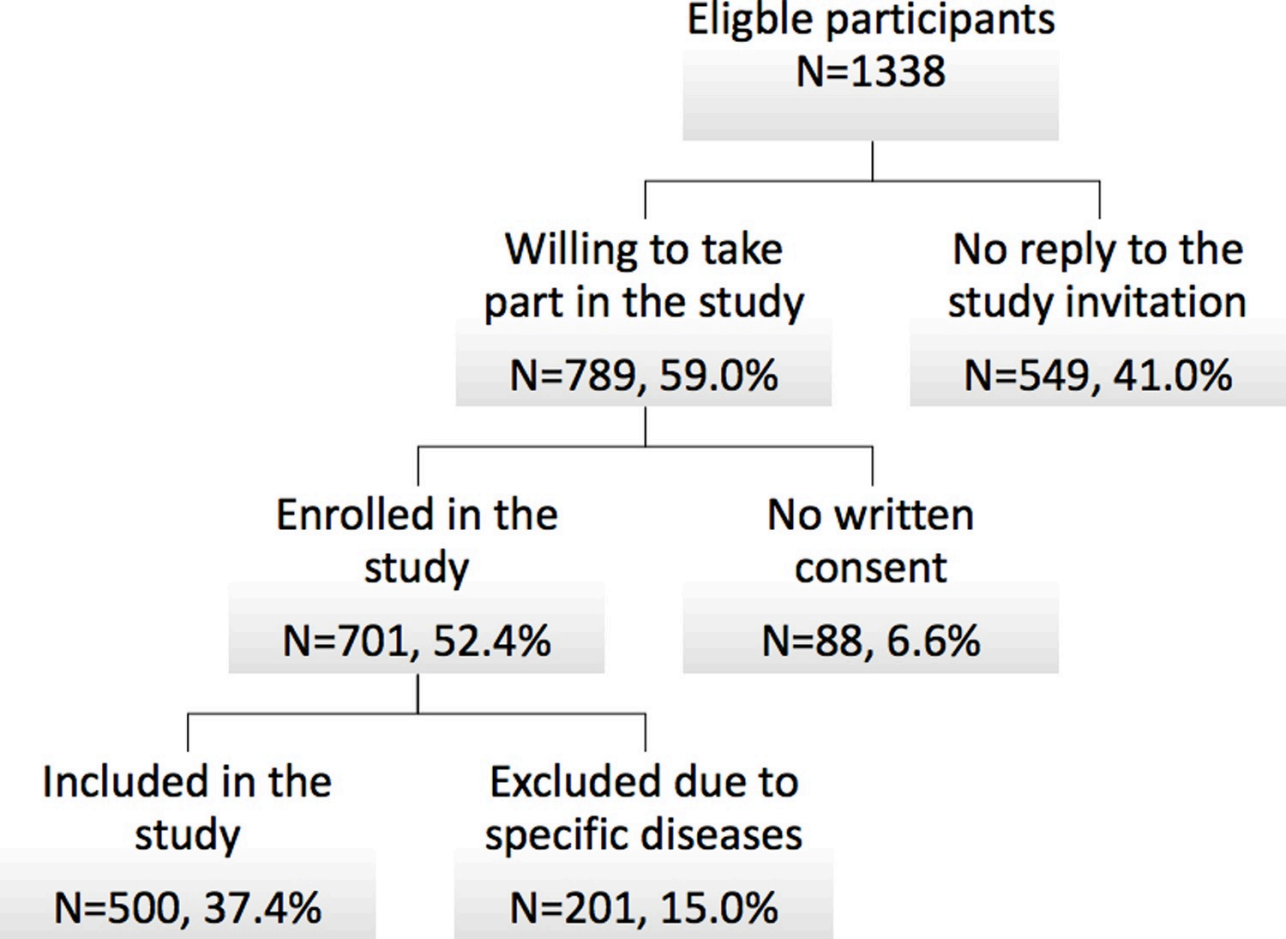
Flow of participants.

The study involved randomly selected participants (n = 1338), both men and women, aged between 65 and 99. However, 41.0% (n = 549) did not respond to the study invitation, 6.6% (n = 88) did not provide written consent to take part in the study and 15.0% (n = 201) were excluded due to the presence of systemic diseases in which periodontal probing, leading to transient bacteremia. Finally, 500 participants were ultimately included in the study (Fig 1).

The inclusion criteria involved age–completed 65 years of age and more, place of living (local resident), the ability to communicate and provision of written consent to participate in the survey. The exclusion criteria included the presence of concurrent systemic diseases, in which periodontal probing, leading to transient bacteremia, might have posed a risk to the patient’s overall health, particularly the subjects diagnosed with cardiovascular diseases (congenital heart diseases, infective endocarditis, heart transplant), blood diseases (thrombocytopenia, haemophilia, von Willebrand disease), viral diseases (B and C type hepatitis, AIDS/HIV) as well as subjects with Multi-Drug Resistant Organisms (MDRO), failure to provide written consent to participate in the survey and the occurrence of mental disorders which render the completion of a questionnaire impossible. All participants involved were obliged to provide written informed consent and completed questionnaire, as well as demonstrate cooperation during the clinical oral examinations. Participants who did not fulfill the inclusion criteria were excluded from the study.

### Sample size estimation

Data on the total number of residents aged 65 and more were derived from Statistics Poland (Demographic Yearbook of Poland 2015) [12]. The sample size was calculated based on the data concerning the number of individuals in this age group who live in the city. With such an assumption, a 95% level of confidence and ±5% margin of error, a minimum sample size consisted of 384 participants [13].

### Ethical permission

The study protocol was approved by the Bioethics Committee of Wroclaw Medical University (permission no. KB 420/2015) under the Declaration of Helsinki. Participation in the study was voluntary and anonymous, and the collected data were treated confidentially.

### Sociodemographic data

Data on sociodemographic data (age, gender, education, living condition, income), self-reported systemic diseases and oral dyscomfort (dry mouth) were collected with use of semi-structured questionnaire.

### Oral examination

Oral examination was performed using artificial light, a plane dental mirror and a ball-ended probe (WHO CPI probe). Coronal and root caries were evaluated according to the World Health Organisation (WHO) criteria, DMFT values and their components. Periodontal health was assessed in individuals (n = 394) who had more than 2 natural teeth by measuring bleeding on probing (BoP) of 6 sites, gingival pocket depth (PD), clinical attachment loss (CAL) [14] and tooth mobility according to Miller’s mobility index [15].

Dental prosthetic status was assessed by taking into account the number of functional tooth units of natural and artificial teeth on implant-supported and fixed prostheses (non-functional tooth units), as well as the information on whether the participants wore partial or complete removable dentures. Based on the clinical oral parameters, a patient’s treatment needs were determined and categorised on a 5-grade scale: no treatment need, preventive treatment, prompt treatment, immediate treatment or referred for comprehensive evaluation or medical/dental treatment (systemic condition).

Moreover, xerostomia (oral dryness) was examined using the Challacombe scale (Clinical Oral Dryness Score, CODS). The level of dryness was categorised based on the number of symptoms observed as mild (1–3), moderate (4–6), or severe (7–10) [16].

Oral examination was conducted by two calibrated examiners. Each examiner was asked to study the same group of 10 patients. The findings were then compared with those of the experienced supervisor. The value of the inter-examiner kappa coefficient amounted to 0.874 while the intra-examiner kappa coefficient was 0.870.

### Quality of life and oral health-related quality of life assessment

QoL was evaluated using three inventories–Euro-Quality of Life (EQ-5D-3L), Oral Health Impact Profile-14 (OHIP-14) and Patient Health Questionnaire (PHQ-9), which were previously validated for the Polish population [17–20].

EQ-5D-3L is a brief, five-item instrument (mobility, self-care, usual activities, pain/discomfort, anxiety/depression) with three response options (no problems, some problems, extreme problems), resulting in potential 243 health states. The obtained health states are converted to a single summary index to reflect a health state according to the preferences of the general population in a country. Sets of EQ-5D index values are constructed at the national level. Moreover, a visual analogue scale (EQ-VAS) was used for the evaluation of a subject’s perception of their own health [17–20].

OHIP-14 consisted of 14 questions related to problems occurring within 1 year: 1) trouble with pronouncing words, 2) worsened taste, 3) pain, 4) discomfort while eating, 5) self-consciousness, 6) emotional tension, 7) unsatisfactory diet, 8) interrupted meals, 9) difficulty with relaxing, 10) embarrassments, 11) irritability, 12) inability to complete everyday tasks, 13) reduced satisfaction with life, 14) complete inability to function. The questions corresponded to 7 dimensions: functional limitation, pain, psychological discomfort, physical disability, psychological disability, social disability and handicap. The frequency of occurrence within 1 year was assessed using the five-point Likert scale: 0 –never, 1 –rarely, 2 –occasionally, 3 –frequently, 4 –very often. All values were summed to calculate the total OHIP-14 score which could vary between 0 and 56; whereby the higher the OHIP-14, the poorer the OHRQoL [21].

PHQ-9 is a questionnaire consisting of 9 basic questions. The answer to each question is scored on a scale ranging from 0 to 3, depending on the frequency of occurrence of a given symptom in the last 2 weeks (3 points indicate the most frequent occurrence of the symptom). The Polish version of PHQ-9, used in the presented study, is available [22]. Diagnosis includes the following severity stages: 0–4—none, 5–9—mild depression, 10–14—moderate depression, 15–19—moderately severe depression, 20–27—severe depression [23].

### Statistical analysis

The obtained questionnaire results and results of clinical trials were statistically analysed using STATISTICA v. 12 software (StatSoft, Inc.). Qualitative, nominal and ordinal variables are shown in tables in terms of sample size (n), set size (N) and proportion (%). The Pearson’s chi-squared (χ^2^) test of independence was used for the assessment of the relationship strength between nominal and ordinal variables. In the case of quantitative variables, arithmetic means (M), standard deviations (SD), medians (Me), lower quartiles (Q1) and upper quartiles (Q3) were calculated and ranges of values from smallest to largest were provided. The normality based on the empirical distributions of the quantitative variables was checked using the Kolmogorov-Smirnov test. Hypotheses concerning the lack of differences between the mean values in two subgroups (women/men) were verified using the non-parametric Mann–Whitney U test of significance. The relationship strength between the EQ-5D-3L, EQ-VAS, OHIP-14, and PHQ-9 psychometric scale scores and sociodemographic and clinical factors was determined by calculating Spearman’s linear correlation coefficient values (rho) and regression coefficient values (b). A t-test based on the Student’s t-distribution with n-1 degrees of freedom was used for verifying the significance of the Pearson’s correlation coefficient. Furthermore, the reliability analysis of each psychometric scale used in the study was conducted by calculating both the Cronbach’s alpha coefficient value and the mean correlation between the items and the final score. In all statistical tests, p<0.05 was taken as the critical value.

## Results

### Characteristics of the participants

The survey involved 500 residents of Wroclaw, aged 65 and older, of both gender. The mean age of the respondents was 74.4±7.4 years. The division of the survey participants according to gender was based on the personal questionnaire and ticking the appropriate box by the respondent. Women made up 64% of the total number of the surveyed group and their mean age was 74.6 ±7.6 years. Most respondents lived together with relatives (male—58.3%, female—64.1%) and did not require assistance with daily activities (male– 90.0%, female-91.3%). Individuals who live alone made up approximately 40% of the total respondents in both subgroups. More females than males had secondary level of education (55.3% vs 43.3%, p = 0.03) and less number of comorbidities (2.2±3.0 vs 1.90±3.0, p = 0.006) (Table 1). However, women more frequently suffered from thyroid diseases and less frequently from genitourinary systemic diseases in comparison to men (respectively, 22.2% vs 4.4%, p<0.001, 15.6% vs 6.3%, p = 0.003) (Table 2).

**Table 1:** Sociodemografic characteristic of participations.

Variables		Males	Females	Difference
		n (%)	n (%)	p value
**Number**		180 (36.0%)	320 (64.0%)	<0.001
**Living with**	alone	75 (41.7%)	115 (35.9%)	>0.05
	other people	105 (58.3%)	205 (64.1%)	
**Living conditions**	home without help	162 (90.0%)	292 (91.3%)	>0.05
	home with help or residence	18 (10.0%)	28 (8.7%)	
**Income**	low	48 (26.7%)	80 (25%)	>0.05
	moderate	71 (39.4%)	155 (48.4%)	
	high	57 (31.7%)	80 (25%)	
	no respond	4 (2.2.%)	5 (1.6%)	
**Education level**	primary	36 (20%)	45 (14.1%)	0.03
	secondary	78 (43.3%)	177 (55.3)	
	higher	66 (36.7%)	98 (30.6)	
		**M±SD**	**M±SD**	
**Age (years)**		73.9±6.9	74.6 ±7.6	>0.05
**Number of comorbidities**		2.2±3.0	1.9±3.0	0.006

M–mean value.

SD—standard deviation.

**Table 2:** Frequency of systemic diseases.

Systemic diseseas	Males		Females		Difference
	n/N	%	n/N	%	p value
**Cardiovascular**	108/180	60.0	211/320	65.9	0.219
**Diabetes**	39/180	21.7	70/320	21.9	0.953
**Respiratory system**	8/180	4.4	29/320	9.1	0.086
**Digestive system**	19/180	10.6	44/320	13.8	0.372
**Blood disease**	2/180	1.1	1/320	0.3	0.295
**Thyroid disease**	8/180	4.4	71/320	22.2	<0.001
**Genitourinary system**	28/180	15.6	20/320	6.3	0.003
**Bone structure**	6/180	3.3	21/320	6.6	0.184
**Rheumatoid diseases**	24/180	13.3	49/320	15.3	0.639
**Cancer**	11/180	6.1	18/320	5.6	0.821

Pearson’s chi-square test of independence

U Mann-Whitney significance test.

### Oral health parameters

#### Dental status

There were no statistically significant differences between men and women in terms of the number of healthy, extracted, and filled teeth (4.4±4.7 vs. 4.8±5.4, 19.0±9.6 vs. 19.1±9.5, 7.4±6.4 vs. 6.3±6.3; p > 0.05) and DMFT value (27.6±4.7 vs. 27.2±5.4; p>0.05). At the same time, the mean number of decayed teeth was significantly higher in men (1.2±2.4 vs. 1.9±3.2, p = 0.034).

#### Periodontal status

There were no statistically significant differences between men and women in terms of tooth mobility (p > 0.05), gingival pocket depth (p>0.05) and the level of clinical attachment loss (p>0.05).

#### Oral hygiene

The mean BoP index (according to Ainamo and Bay, 1975) was 53.0% ± 100.0 in women and 60.08% ± 100.0 in men, indicating poor oral hygiene.

#### Oral dryness

Mild xerostomia was diagnosed significantly more frequently in women (36.9% vs. 25.5%; p = 0.076). The most typical sign of oral dryness was dental mirror sticking to the buccal mucosa, which also occurred significantly more frequently in the female group (30.6% vs. 18.3%; p < 0.01).

#### Prosthetic status

Women did not differ significantly from men in terms of the frequency of use of removable dentures (p> 0.05). Moreover, the mean number of natural teeth in the maxilla (including teeth covered by prosthetic crowns, replaced by pontics and implants) in contact with the mandibular (lower) teeth was similar in both genders (Table 3).

**Table 3:** Oral health parameters.

Oral health parameters	Males	Females	Difference p value
	M±SD	M±SD	
**Number of natural teeth**	13.0 ± 9.5	13.0 ± 9.6	0.954
**DMFT**	27.2 ± 5.4	27.6 ± 4.7	0.826
**DT**	1.9 ± 3.2	1.2 ± 2.4	0.034
**MT**	19.1 ± 9.5	19.0 ± 9.6	0.954
**FT**	6.3 ± 6.3	7.4 ± 6.4	0.073
**Number of teeth with wear**	6.5 ± 10.5	5.6 ± 9.0	0.321
**Number of occluding teeth**	4.6 ± 9.0	4.8 ± 9.0	0.741
**Bleeding on Probing (% sites)**	60.8 ± 100.0	53.0 ± 100.0	0.113
**Pocket Depth**	3.7 ± 4.1	3.7 ± 4.1	0.579
**CAL (mm)**	4.2 ± 5.0	4.2 ± 5.0	0.492
**Movable teeth number**	0.8 ±0.0	0.6 ±0.0	0.209
	**n/N %**	**n/N %**	
**Partial movable prothesis in maxilla**	50/180 27.8%	104/320 32.5%	0.480
**Partial movable prothesis in mandible**	44/180 24.4%	86/320 26.9%	0.856
**Total movable prosthesis in maxilla**	45/180 25.0%	80/320 25.0%	0.917
**Total movable prosthesis in mandible**	36/180 20.0%	77/320 24.1%	0.469
**Oral dryness**	46/180 25.5%	118/320 36.9%	0.076

The chi-square statistic with Yates correction.

#### Treatment needs

When assessing treatment needs in the studied groups, it was found that men were significantly more likely to have high treatment needs (36.1% vs. 26.9%) and they required urgent dental treatment (7.2% vs. 2.8%) (Table 4).

**Table 4:** Treatment needs.

Code	Intervention urgency	Males		Females		Difference p value
		n/N	%	n/N	%	
0	no treatment need	13/180	7.2	39/320	12.2	0.011
1	preventive treatment	89/180	49.4	185/320	57.8	
2	prompt treatment	65/180	36.1	86/320	26.9	
3	immediate treatment	13/180	7.2	9/320	2.8	
4	referred	0	0	1/320	0.3	

### Quality of life

There were no statistically significant differences between genders in terms of QoL evaluated by EQ-5D, EQ-5D VAS or OHIP-14 scores (0.832±0.194 vs 0.855±0.197, p = 0.089; 67.9±10.9 vs 66.1±18.6, p = 0.174; 7.2±12.9 vs 8.5±14.0, p = 0.28). Only women had higher severity of depressive symptoms measured using the PHQ-9 questionnaire (4.0±4.1 vs. 2.8±3.8; p<0.001) (Table 5).

**Table 5:** Mean scores of inventories.

Gender		Males	Females	Difference
				p value
**EQ-5D index**	*M* ± *SD*	0.832 ± 0.194	0.855 ± 0.197	0.089
	*Me* [*Q*_1_; *Q*_3_]	0.87 [0.74; 1.00]	0.89 [0.82; 1.0]	
	*Min*—*Max*	0.09–1.00	0.09–1.00	
**EQ-5D VAS**	*M* ± *SD*	67.9 ± 10.9	66.1 ± 18.6	0.174
	*Me* [*Q*_1_; *Q*_3_]	70 [50; 80]	70 [50; 80]	
	*Min*—*Max*	5–100	20–100	
**OHIP-14**	*M* ± *SD*	7.2 ± 12.9	8.5 ± 14.0	0.280
	*Me* [*Q*_1_; *Q*_3_]	0 [0; 8]	1 [0; 10]	
	*Min*—*Max*	0–56	0–56	
**PHQ-9**	*M* ± *SD*	2.8 ± 3.8	4.0 ± 4.1	<0.001
	*Me* [*Q*_1_; *Q*_3_]	2 [0; 4]	3 [0; 7]	
	*Min*—*Max*	0–17	0–21	

M–mean value

SD—standard deviation

Me—median

Q1—lower quartile (25th percentile)

Q3—upper quartile (75th percentile)

Min—the smallest value

Max—the largest value

p–significance level according to the non-parametric U Mann-Whitney test.

When considering the relationships between scores of the inventories used, a positive correlation was observed between the OHIP-14 and EQ-5D (rho = 0.242, p< 0.001), EQ-5D VAS (rho = -0.123, p = 0.01) and PHQ-9 (rho = 0.334, p< 0.001).

### Linear regression analyses

In the case of the univariate analysis, the predictors of the better QoL assessed using the EQ-5D questionnaire included female gender, age below 75, independence in daily life, a low number of comorbidities (less than 2), as well as high or moderate income and higher or secondary level of education. As regards the oral health parameters, the predictors of the better QoL using the EQ-5D questionnaire included low OHIP-14 scores, lack of oral dryness, no treatment need or preventive treatment need.

According to the results of the multivariate linear regression analysis between QoL assessed using the EQ-5D questionnaire and significantly differentiating parameters, the independent predictors which significantly affect high QoL scores on the EQ-5D scale included female gender, age below 75, living at home without required assistance, mild depression assessed using the PHQ-9 questionnaire, high or medium income, no oral treatment needs (Table 6). The fit of the proposed model to the measurement results is satisfactory: F(7.492) = 38.9, p < 0.001; the multiple correlation coefficient of the model is R = 0. 597, the mean of squared residuals is MSE = 2.70.

**Table 6:** Predictors of QoL assessed by EQ-5D questionnaire–values of linear regression coefficients.

Predictors	Univariate regression		Multivariate regression	
	b	p	beta	p
Female	0.188	0.049	**0.284**	**<0.001**
Age lower than 75 years	0.582	<0.001	**0.297**	**<0.001**
Living home without help	1.226	<0.001	**0.845**	**<0.001**
Income medium or high	0.556	<0.001	**0.284**	**0.001**
Number of systemic diseases	0.600	<0.001	**0.422**	**<0.001**
No treatment or preventive treatment need	0.403	<0.001	**0.232**	**0.004**
OHIP-14 score	0.022	0.001	-	> 0.05
PHQ-9 score	0.197	<0.001	**0.147**	**<0.001**

## Discussion

Population ageing is a global phenomenon. With advances in medicine and prolonged life expectancy, the proportion of the elderly population will continue to grow worldwide. According to the United Nations prognosis, the number of the elderly population (aged 65 and more) between 2019 and 2050 will grow worldwide approximately by 120% (from 9.5% to 15.9%), while in Europe and Northern America approximately by 48% (from 18.0% to 26.1%) [24]. Conventionally, the elderly population is defined as people aged 65 and more [25]. In Poland, the proportion of the elderly population in 2008 was 13.5% and 10 years later it increased up to 17.5% [13]. In subsequent years, compared to 2010, the number of people aged 80 and older will increase significantly in Poland (2010–4.46%, 2060–13.08%). Similar demographic changes will occur in 27 EU countries (2010–4.66%, 2020–5.70%, 2060–12.13%) [5, 7, 26]. The characterization of the sample revealed a predominance of women (64.0%), a result that is similar to that observed in other studies [27–29]. This fact may be explained by the longer life expectancy of women, which highlights the feminization of old age.The increase in the elderly population caused by prolonged life expectancy requires special attention to various health-related variables that influence their QoL [30]. Old age is related to an increase of chronic diseases and disabilities, oral and dental problems as well as some physiological changes. The main oral diseases–dental caries and periodontal diseases are cumulative with age, therefore their severity and sequelae increase leading to loss of natural teeth and functional impairment of oral masticatory system. It has been evidenced by the close bi-directional relationship between oral and overall health, as well as by an impact of oral health on QoL. That is why the oral health-related quality of life, OHRQoL, is an integral part of overall health and psychological well-being. It is a multidimensional construct that includes a subjective evaluation of an individual’s oral health, functional well-being, emotional well-being, expectations and satisfaction with care, and sense of self [31].

The available standardised questionnaires, measuring QoL including health status, allow choosing an adequate tool for the psychometric assessment of a specific research group. Health-related quality of life has been frequently used by EQ-5D-3L to measure of health for clinical and economic appraisal. It can be used not only to describe the subject health at one point in time, but also changes in health over time, for example before and after treatment. The EQ-5D provides an index value (‘utility’) for health status and a simple descriptive profile, a self-report visual analogue scale (EQ VAS) in population health surveys. Population norms differs in terms of age, gender and country [17, 32].

The value set of EQ-5D-3L based on the time trade-off (TTO) for Polish general population was described by Golicki [33]. Our data based on the participants at average age of 74.4 years showed some lower values of the EQ-5D index in males than females. Whereas Polish normative data for pointed on some lower these values in women in comparison to men in the age groups of 65–74 and 75 and over (0.802 vs 0.831 and 0.712 vs 0.767, respectively) [33].

Self-reported health condition according to EQ VAS in the studied participants was a little higher in men then women which is consistent with data for the elderly Polish population. In the age group of 65–74 among men EQ VAS was 64.8 and in women 61.2, and end the age of 75 and over 55.0 and 53.2, respectively [33]. However, the analysis of EQ-5D population norms for 20 countries showed that in some countries EQ VAS values are higher in males then females in the both age groups (for instance France, Germany, Hungary, Slovenia) and in others only in one age group (for example Denmark, Netherlands) [32].

There are numerous questionnaires available for measuring OHRQoL, however, the most frequently used questionnaires include Geriatric Oral Health Assessment Index (GOHAI) and Oral Health Impact Profile (OHIP) containing 49 or 14 items (OHIP-49, OHIP-14). In the literature, the studies concerning gender differences in terms of oral health showed that women exhibited more positive oral health attitude and oral health behaviour (tooth brushing frequency; using dental floss; regular dental visits) [27–29]. This confirms the conclusions obtained in the present study in which the male respondents had greater oral treatment needs compared to the group consisting of older women. The present study found that men had a significantly higher mean number of decayed teeth, but the assessment of periodontal parameters of the elderly of both gender showed no statistically significant differences. Similarly Marya et al. [34] observed no significant differences in the periodontal status among men and women. There were no statistically significant differences between both gender in terms of the prevalence of gingival bleeding, periodontal PD, and tooth mobility in the surveyed population. In contrast, some studies showed higher caries rates in women [35] and higher prevalence of gingivitis and periodontitis in men [36].

Oral diseases adversely affect the overall health and QoL. A well-functioning dentition is crucial for the elderly since it supports main functions such as eating, speaking, smiling and socialising. The consequences of untreated dental caries and periodontal diseases are related to overall health and psychological well-being [37]. The results of the presented study showed that low OHIP-14 scores and female gender were predictors of QoL assessed using the EQ-5D questionnaire. The null hypothesis was rejected, as there were differences in some studied parameters between males and females. These study results are in contrast to Marya et al.’s [34] who observed that OHRQoL was better in men. The study conducted in north-east urban area of Poland and involving a similar age group of older people showed a correlation between OHIP-14 for both gender and age, several extracted teeth, DMF, several present teeth [38]. A study involving British 65-year-olds found that gender was not significantly related to OHIP-14 [39]. Saintrain et al. [40] and Chen Yu-Fen [41] observed no significant differences in OHIP-14 scores for both gender either. The similar results were obtained by Saxena et al. [42] who found no significant differences between men and women in individual OHIP -14 domains. Dallasta et al. [43] who studied women aged 60 and more, recorded the highest values for "physical pain" = 1.98±1.41, "psychological distress" = 1.83±1.92 and "physical disability" = 1.63±1.53.

In our study, women were found to have higher severity of depressive symptoms measured using the PHQ-9 questionnaire. Besides, the predictors which significantly affect HQoL scores on the EQ-5D scale were identified as low levels of depression assessed using the PHQ-9 questionnaire. The Patient Health Questionnaire-9 is widely used as a screening instrument for depression detection as it can be self-administered. The elderly age and loading by chronic physical diseases are regarded as possible risk factors for depressive disorder [44]. In the European Union countries 6.8% of the adult population (18 years and over) experiencing depressive symptoms, in Poland 5.1% [45]. The results of the presented study showed a moderate correlation coefficient between the sum of the PHQ-9 scores and the EQ-5D index (rho = 0.334, p<0.001) which confirmed an impact of the depression presence on general quality of life. The similar relationship was obtained in adult Korean population [46]. When it comes to the PHQ-9 scale, other Polish studies involving a younger age group, with a mean age of 41 years, found no significant differences in scores obtained by 54 women and 45 men [19]. In contrast to the above-mentioned studies, Shin et al.’s [46] study involving a Korean population of men and women of different ages found that mean PHQ-9 scores were significantly lower in men in all age groups and there was an upward trend in the mean PHQ-9 scores as age increased in studied age groups. The study results concerning Korean women aged 19 and older also showed a correlation between PHQ-9 and EQ-5D, which coincides with the results of the present study.

Several limitations need to be taken into account in a discussion concerning the results of the present study. Firstly, the study was conducted using a self-reported questionnaire to report data such as EQ-5D, PHQ-9, OHIP-14, which might have led to identification bias. However, some studies proved that the questionnaire could be used as a valid and reliable method. Secondly, the use of survey data did not allow us to explain temporal relationships nor to show inferences on causality. Thirdly, when analysing the obtained results, it is essential to bear in mind a possible self-selection error of the study participants–those who participated in the study were concerned about their dental problems or were aware of those problems and they were looking for help. Moreover, some persons may have refused to participate in the study due to their dental fear. Another interfering factor was related to the use of exclusion criteria. The study was limited by the exclusion of patients with concurrent systemic diseases in whom periodontal probing, leading to transient bacteremia, might have posed a risk to their overall health. That exclusion criterion involved many patients in this age group. Finally, the exclusion of patients with a mental disorder might have constituted another interfering factor.

## Conclusion

A study conducted on a group of the Polish elderly population showed:

1. Significant differences in education and income levels between men and women, which were found to be positive predictors for the evaluation of QoL using the EQ-5D questionnaire.
2. Male respondents suffered from more systemic diseases compared to women, as well as they had greater oral treatment needs and a statistically more decayed teeth.
3. Depression and xerostomia were most frequently diagnosed in women. Low levels of depression assessed using the PHQ-9 questionnaire were related to high QoL scores on the EQ-5D scale.
4. The independent predictors which significantly affect the high QoL scores on the EQ-5D scale were found to be female gender, age below 75, high or middle income, independence in daily life, a low number of comorbidities, lack of oral treatment needs.

In recent years, we have been observing an increasing number of the elderly in society, especially older single woman also known as feminization of old age. That indicates the need to identify activities improving quality of life people in post- production age. Active ageing means growing old in good health accompanied by active participation in social life, professional fulfillment and independence in daily tasks.

## List of abbreviations

QoL: Quality of Life
OHRQoL: Oral Health-Related Quality of Life
EQ-5D-3L: Euro-Quality of Life
PHQ-9: Patient Health Questionnaire-9
OHIP-14: Oral Health Impact Profile- 14
DMFT: Decayed Missing Filled teeth index
DT: Number of Decayed Teeth
MT: Number of Missing Teeth
FT: Number of Filled Teeth
BoP: bleeding on probing
PD: gingival pocket depth
CAL: clinical attachment loss
SES: socioeconomic status
